# Plan B for Stimulating Stem Cell Division

**DOI:** 10.1371/journal.pgen.1003117

**Published:** 2012-11-15

**Authors:** M. Kathryn Barton

**Affiliations:** Department of Plant Biology, Carnegie Institution for Science, Stanford, California, United States of America; The University of North Carolina at Chapel Hill, United States of America

Plant development relies on two kinds of coordinated regulatory inputs to generate an optimal plant body. First are inputs regulating the spatial organization of cells in the plant. These “hardwired” inputs are invariant between individuals and their actions are buffered from the environment. Second are variable inputs that modify the development of tissues to optimize growth for given conditions of water, gravity, nutrients, and light. Defining these pathways and understanding how they work together is a major challenge for plant biologists. Work by Turner and colleagues in this issue of *PLOS Genetics* [1] moves us a step closer by elucidating a link between two pathways that control proliferation of a stem cell population that produces vascular cells. These two pathways are a receptor–ligand pathway, which represents the first type of hardwired machinery, and the ethylene signaling pathway, which traditionally has been considered an environmentally dependent pathway.

In growing plants, stem cells at the tips of roots and shoots add new cells to the plant body. In shoots these cells generate new organs, leaves, and stem sections, each segment of leaf and stem adding additional length to the plant body. This can lead to very long branches. However, in order for plants to reach a significant size, they must add cells to their girth as well. Adding girth allows the plant to support branches and lets these expand the canopy where newly made leaves can compete for sunlight.

**Figure 1:**
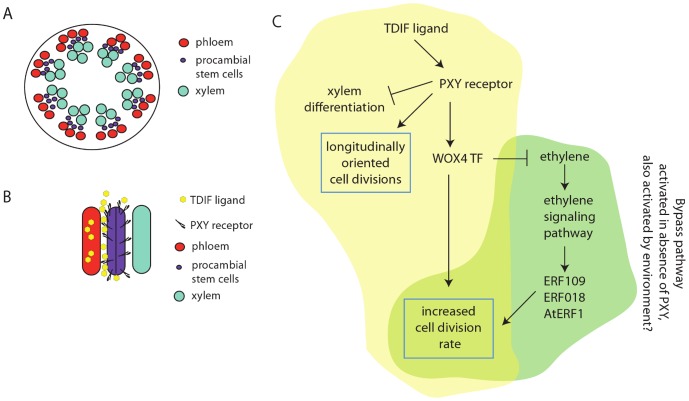
Role of the TDIF/PXY ligand receptor and ethylene signaling pathways in the promotion of cell division in the cambial stem cells of *Arabidopsis*. (A) Schematic of stem cross section. In dicots such as *Arabidopsis* the phloem (red) and xylem (blue) form concentric rings near the surface of the plant. Procambial stem cells (purple) are located between them. (B) Longitudinal schematic of phloem, procambial, and xylem cells. The TDIF peptide is made in the phloem and acts non–cell autonomously on the PXY receptor-expressing procambial stem cells. The asymmetric signal from TDIF on PXY acts to orient cell division longitudinally such that daughter cells are long and slender and such that stem cell descendants are pushed either outward toward phloem or inward toward the xylem, thus maintaining the organization of these tissues. TDIF, acting through PXY, suppresses xylem differentiation and promotes cell division. Increasing the rate of cell division requires the WOX4 transcription factor. (C) The TDIF signal acts on PXY to activate WOX4, to promote cell division. The TDIF effects on the orientation of cell division and on the suppression of xylem differentiation do not go through WOX4. In this model, TDIF/PXY also represses ethylene production and thus the alternative branch of the pathway. In the absence of the PXY protein, ethylene levels are increased resulting in an increase in ERF109, ERF018, and AtERF1 levels and stimulation of cell division.

Girth is added through the action of a second source of stem cells. These stem cells are located in a ring within the stem or trunk of a tree, where they generate new vascular cells (Figure 1A). In the stem of *Arabidopsis*, these are called procambial cells. The procambial stem cells are sandwiched between the two vascular cell types they give rise to: phloem cells (toward the outside of the plant), the carriers of sugars from the leaves to roots, fruits, and other “sink” organs; and xylem cells (toward the inside of the plant), the carriers of water and minerals. Procambium is used to denote the stem cells in vascular bundles of newly formed organs. In plants such as trees that show persistent lateral growth, the procambium gives rise to a more substantial, continuous ring of stem cells called a cambium. *Arabidopsis*, while not a perennial, and certainly not a tree, nevertheless exhibits secondary growth and a well-developed cambial stem cell population in the hypocotyl, the short stem below the rosette.

The position of the procambium between the two types of descendant cells is critical to its production of organized phloem and xylem strands. Regulated orientation of cell divisions within the procambium maintains this organization as newly generated cells are fed into the differentiation pathways. Cell divisions in the long, narrow progenitor cells are oriented along the long axis of the stem (Figure 1B). Since most plant cell division planes cut across the narrowest dimension of the cell, orienting new walls such that they span the longest dimension likely requires specialized machinery controlled by specialized regulators.

Environmental cues affect the activity of cambial stem cells. In trees, the vascular cambium goes through cycles of activity and inactivity with seasons. In winter the cambium is dormant, but it becomes active again during summer, resulting in the characteristic annular rings of wood. Gravity also regulates cambial growth: when trees lean, the cambium on the upper side of the trunk grows at a different rate from the lower side to generate structural support (i.e., “tension wood”) [2].

In *Arabidopsis*, the tracheary element differentiation inhibition factor/CLE41 (TDIF/CLE41) peptide ligand is secreted from the phloem and interacts with the TDIF RECEPTOR/PHLOEM INTERCALATED WITH XYLEM (TDR/PXY) membrane receptor kinase expressed in adjacent cambial stem cells (Figure 1B) [3]. This signal accomplishes three things. First, it stimulates cell division within the cambium. To do this, it requires the downstream transcription factor WOX4. Second, it prevents stem cells in the cambium from becoming xylem cells. Third, it regulates the orientation of cell divisions. The latter two steps do not require WOX4 action [4], [5].

Regulation of cell division orientation in the procambium requires the polar production of TDIF peptide [4]. If TDIF peptide is produced on both sides of the cambium, or only on the xylem side, cell division planes in the procambial cells become highly irregular. Thus, the tissue-specific synthesis of TDIF (in the phloem) and the detection of its asymmetric distribution (in the procambium) are part of an invariant developmental pathway that produces spatially organized vascular strands.

The phenotypes caused by overexpressed TDIF peptide were eliminated in *pxy* mutants, indicating that the TDIF signal requires the PXY receptor to act. However, surprisingly, loss-of-function *pxy* mutants exhibited only a mild decrease in procambial cell numbers. This suggests that the plant possesses a “plan B” to stimulate procambial stem cell division. In their new work, Turner and colleagues [1] identify the bypass mechanism as signaling through the gaseous hormone ethylene.

The first clue that ethylene mediates the bypass pathway came when Etchells et al. found increases in mRNA abundance for some members of the APETALA2/ETHYLENE RESPONSE FACTOR (AP2/ERF) family of transcription factors in *pxy* mutants. Since *AP2*/*ERF* gene family expression is elevated in response to ethylene and mediates ethylene response, Etchells et al. reasoned that ERFs might compensate for the absence of PXY. Indeed, when loss-of-function mutations in the AP2/ERF factor genes *ERF109*, *ERF018*, and *At1ERF* were combined with mutations at the *PXY* locus, the resulting *pxy erf* double mutants had significantly reduced numbers of cambial stem cells. This demonstrates that the *AP2*/*ERF* genes act in a pathway that is functionally redundant to the *PXY* pathway.

Etchells et al. also show that an increase in ethylene stimulates cell division in procambial stem cells. Moreover, ethylene upregulates these particular *AP2*/*ERF* genes. Finally, when *pxy* mutations are combined with mutations that disrupt the ethylene signaling pathway upstream of the ERF factors, procambial cell numbers are significantly decreased. Thus, the bypass pathway requires ethylene to function.

Yet another link to the ethylene pathway exists in this system: *ACS6* mRNA levels (ACS is an enzyme that catalyzes ethylene biosynthesis) are upregulated in *pxy* mutants. These findings suggest the existence of an ethylene-based bypass pathway that is normally off but becomes activated when *PXY* activity is low or missing (Figure 1C). The *TDIF–PXY–WOX4* pathway normally keeps ethylene levels low and, through an as yet unknown pathway, stimulates cell division in procambial stem cells. In the absence of *PXY*, ethylene increases and stimulates the production of the *ERF109*, *ERF018*, and *AtERF1* transcription factors, which in turn activate procambial stem cell division.

These findings are consistent with, and to some degree inspired by, earlier findings on the role of ethylene in promoting cell division in poplar trees [6]. Blocking ethylene perception, either chemically with an ethylene antagonist or genetically by introducing a dominant negative ethylene receptor mutation, blocked cell division in the cambium. Moreover, excess ethylene increased the number of cell divisions in the cambium. Significantly, in woody plants that lean to one side, blocking ethylene action resulted in a failure to form tension wood, indicating that the mechanical/gravitational changes sensed by the cambium in poplar require ethylene signaling.

In summary, a spatially organized, tissue-specific program is established by TDIF ligand synthesis in one pole of the vascular strand and asymmetric TDIF sensing by the corresponding PXY receptor present in the procambial stem cells. It is likely that this pathway (highlighted in yellow in Figure 1C) is responsible for directing the pattern of early cell divisions in the procambial stem cells of the vascular bundles. A second pathway, the ethylene signaling pathway (highlighted in green in Figure 1C) can also stimulate procambial cell divisions. This pathway is normally “OFF” when PXY functions; this “OFF” state may limit procambial cell divisions during critical developmental stages when new vascular strands are established and form connections with established veins. It remains to be seen under what conditions the alternative ethylene branch of the pathway is activated. Given the ethylene-dependent stimulation of tension wood formation by gravity in poplar, it is tempting to speculate that there are as yet undiscovered environmental inputs for this bypass pathway. If this is the case, the two pathways may provide a prototypic example of how invariant pathways that specify the spatial organization and division activity of cells are integrated with environmental cues that further tune the plant body to its environment.
